# Men’s perceptions of HIV care engagement at the facility- and provider-levels: Experiences in Cote d’Ivoire

**DOI:** 10.1371/journal.pone.0211385

**Published:** 2019-03-21

**Authors:** Natalie Jean Tibbels, Zoé Mistrale Hendrickson, Danielle Amani Naugle, Abdul Dosso, Lynn Van Lith, Elizabeth C. Mallalieu, Anne Marie Kouadio, Walter Kra, Diarra Kamara, Patricia Dailly-Ajavon, Adama Cisse, Kim Seifert-Ahanda, Sereen Thaddeus, Stella Babalola, Christopher J. Hoffmann

**Affiliations:** 1 Johns Hopkins Center for Communication Programs, Johns Hopkins Bloomberg School of Public Health, Baltimore, Maryland, United States of America; 2 Johns Hopkins Center for Communication Programs, Johns Hopkins Bloomberg School of Public Health, Abidjan, Côte d’Ivoire; 3 Sociology Department, Félix Houphouët-Boigny University in Cocody, Abidjan, Côte d’Ivoire; 4 Sociology Department, Alassane Ouattra University, Bouaké, Côte d’Ivoire; 5 United States Agency for International Development, Washington, D.C., United States of America; 6 United States Agency for International Development, Abidjan, Cote d’Ivoire; 7 School of Medicine, Johns Hopkins University, Baltimore, Maryland, United States of America; University of South Florida, UNITED STATES

## Abstract

Men in sub-Saharan Africa have lower rates of HIV testing and are less likely to initiate treatment compared to women. Service delivery dimensions are a key factor in facilitating engagement along the HIV treatment continuum for men and women, yet male specific overall perceptions of the service delivery environment have received little attention in West Africa. This study draws on qualitative data collected in Côte d’Ivoire to explore provider-level and structural factors affecting men’s engagement in HIV testing and treatment through interviews and focus group discussions conducted with health workers and men living with HIV (some on ART) or whose HIV status was unknown. Factors influencing decisions to test or initiate treatment were considered in terms of perceived benefits and costs. Men described costs at the interpersonal (client-provider) level, such as unwanted disclosure or stigma, which were weighed against the potential for social support and clinical guidance. Likewise, fear of unwanted disclosure operated at the facility level, as the layout of facilities sometimes grouped clients living with HIV together. Notably, the benefits men described from engaging in HIV testing and care all operated at the interpersonal level and none at the facility level. In light of the fact that provider- and facility-level factors influenced the perceptions and experiences of men along the treatment continuum, we offer recommendations to reduce barriers to testing and engagement in care related to service delivery.

## Introduction

Côte d’Ivoire is one of 35 priority countries identified by the World Health Organization that account for 90% of new HIV infections globally and have been giving specific transmission, testing, and treatment targets [1]. Côte d’Ivoire has one of the highest HIV-prevalence rates in West Africa with 2.8% of adults of reproductive age (15–49) living with HIV [2]. Though AIDS-related deaths have declined considerably in the past 15 years, universal access to HIV treatment is considered necessary to end the epidemic[3,4]. To accelerate the global decline in AIDS-related deaths, the Joint United Nations Programme on HIV/AIDS established the 90-90-90 targets: by 2020, 90% of people living with HIV will know their HIV status, 90% of people diagnosed with HIV will receive sustained antiretroviral therapy (ART), and 90% of people receiving ARTs will achieve viral suppression [3]. If the 90-90-90 target is met, AIDS related deaths globally are expected to decline from one million in 2016 to fewer than 500,000 in 2020 [1] and new HIV infections in Côte d’Ivoire could be reduced by 50% by 2030 [5]. Throughout sub-Saharan Africa, a higher proportion of women than men undergo HIV testing and a higher proportion of women living with HIV have been diagnosed and are on ART [6]. For example, in Cote d’Ivoire, 60% of women living with HIV aged 15 or older compared to 29% of their male counterparts are on ART [2]. In addition, following diagnosis, men are less likely to link to care, achieve viral load suppression, or survive[7–9]. Men have particularly low levels of testing, with 75% of men (vs. 62% of women) unaware of their HIV status [10]. In light of the difference in outcomes between men and women, population-specific approaches to improving outcomes among men are warranted, including strategies to improve the continuum of HIV care among men [11].

Reaching the 90-90-90 targets requires increasing demand for and access to HIV testing and treatment services. The government of Côte d’Ivoire began implementing Test and Start in early 2017, eliminating eligibility criteria for accessing ART. ART is and has been provided free of charge in public sector facilities. However, tailored strategies are needed to increase those accessing HIV testing and treatment services, including services geared to priority populations with lower engagement in HIV-related services such as men.

Prior research suggests that the reasons for the disparity between men and women in seeking HIV services are related to both gender norms and service-related factors. A longitudinal qualitative research study embedded within a trial of Test and Start in East Africa found the demands of work, cultural associations of medical spaces and care-seeking as a female space or activity, as well as fear of the consequences of an HIV-positive diagnosis to be barriers to HIV-testing among men [12]. A framework developed to describe engagement with the HIV care continuum in low- and middle-income countries (LMICs) suggests that individuals unconsciously weigh perceived values or benefits associated with care-seeking (e.g. social support or feeling healthy) against perceived costs (e.g. stigmatizing treatment or inefficient services) [13]. Value/cost factors influencing decision-making operate at each level of the socioecological model: from the individual, interpersonal, facility, and community up to the policy domain. The value/cost model also suggests that many of these “decisions” occur unconsciously and are based on cognitive biases described in the field of behavioral economics [13]. Within public health, values are more commonly termed motivating factors, facilitators, or benefits, and costs are usually framed as barriers; the benefit and cost language captures the complex conscious and sub-conscious calculations that occur in decision-making about HIV service uptake. In general, these individual- and community-level factors for both men and women have been the focus of considerable research [14–17]. However, male perceptions of factors operating at the health facility level warrant further study in light of men’s lower testing rates and engagement in care in sub-Saharan Africa.

We focused on perceived service-related benefit and cost factors affecting engagement with HIV services among men in Côte d’Ivoire. Service-related costs and benefits can be separated into provider- factors and facility infrastructure factors. At the provider level benefit and cost is influenced by the extent of trust in relationships with health workers, the quality of counseling, and whether confidentiality is protected [14,18] Facility-level factors include: complex scheduling practices, long wait times, stock outs, lack of private counseling rooms, and accessibility [19–21] In this study, we focus on provider and the health facility costs (barriers associated with the facility, apart from those related to interactions with the provider) and benefits as perceived by men. Specifically, we sought to understand perceptions regarding accessing care among men living with HIV (MLHIV) on treatment, MLHIV not on treatment, and men whose HIV status was unknown to investigators.

## Methods

To explore provider- and facility-level factors that affected men’s engagement along the HIV continuum of care, we explored men’s perspectives from data collected as part of a larger formative qualitative study. As part of that study, men’s perceptions and values were sought through in-depth interviews (IDIs) and focus-group discussions (FGDs) conducted in three urban areas in Côte d’Ivoire: Abidjan, Bouaké, and San Pedro. The research protocol and instruments were approved by the Johns Hopkins Bloomberg School of Public Health (JHSPH) Institutional Review Board [IRB#00007374] and the local ethics committee (Comité National d’Ethique de la Recherche) in Côte d’Ivoire. Written informed consent was obtained from all participants prior to data collection.

### Participants

IDIs and FGDs were conducted in fall 2016 with men between 25 and 49 years of age who were either living with HIV or whose HIV status was unknown to investigators. We used purposive sampling to recruit participants using recruitment scripts with support from local non-governmental organizations (NGOs). Twenty-eight IDIs were conducted with MLHIV in treatment (15) and MLHIV not in treatment (13). In addition, 45 IDIs and 28 FGDs were conducted with men whose HIV status was unknown to investigators.

### Recruitment and data collection

Participants were recruited using venue-based sampling (in person) using recruitment scripts through NGOs implementing Brothers for Life groups, community social groups for men, and workplaces. Brothers for Life is a community-based program engaging adult men in workshops covering HIV and other health-related topics, implemented with USAID funding by the Johns Hopkins Center for Communication Programs. Snowball sampling was used in particular cases, in cases where community leaders were willing to invite community members to participate. The recruitment script described the goal of the research study as informing community programs and health messages about HIV testing and treatment in Côte d’Ivoire. There was no prior relationship between researchers and participants. IDIs and FGDs were conducted at NGO offices or health facilities in locations where participants’ privacy and confidentiality could be ensured. Before the interview or discussion began, the facilitator read an information note detailing the purpose, procedures, risks, and benefits of the study, and then invited individuals to sign a consent form. The consent form was collected; the participant retained the information note. A team of Ivoirian researchers with doctorates in sociology and experience with qualitative research, led by authors AK and WK, conducted the interviews. DN trained the data collection team in the study protocol and research ethics. Men were interviewed once in each of the three urban areas by male interviewers in light of the sensitivity of the interview topics. Researchers conducted interviews with participants in private locations with no one else present. On average, IDIs lasted around 45 minutes and FGDs around an hour and fifteen minutes.

IDI and FGD guides provided by the authors focused on men’s values and aspirations; knowledge, beliefs, and perceived social norms about HIV testing and treatment; and experiences engaging in the HIV continuum of care (e.g. HIV testing, diagnosis, or treatment). Interviews were conducted, audio-recorded, and transcribed word-for-word in French for the purposes of analysis. The data collection team kept field notes, which informed a field report that was prepared immediately after the end of data collection. Twenty percent of transcripts were double-checked to assess consistency with audio recordings.

### Analysis

Data coding and analysis were conducted in French using Atlas.ti. [22] The research team conducted a thematic content analysis using both inductive and deductive approaches to develop and refine the codebook. We developed an initial structured codebook that was informed by existing literature on the multi-level barriers to HIV testing and treatment and social and behavior change theory. [13,23] Codes were also developed inductively based on emergent themes from the transcripts. An initial set of transcripts was coded using the preliminary codebook. Following open coding, codes relevant to similar themes were linked together or merged in Atlas.ti. New codes and sub-codes identified during the process were added to the codebook and the initial set of transcripts was re-coded with the revised codebook. The codebook was iteratively developed and definitions refined to ensure shared understanding of meaning and application. A team of five researchers who were all bilingual in French and English coded the transcripts, with twenty percent of interviews double-coded to ensure consistency. Coders resolved discrepancies through discussion or consultation with a third coder. The development and finalization of the codebook was an iterative process based on multiple rounds of data coding, reflection, and revision. Memos were drafted in English during the coding phase to encourage researcher reflexivity and to allow for engagement of non-French speaking team members in the analysis process. Saturation in themes was discussed during daily debrief meetings throughout data collection and again during data coding and analysis; we assessed informational saturation during iterative coding, memo-ing, and discussions of the research team.

Codes, and their associated quotations, related to provider and health facility factors were reviewed and analyzed for the purposes of this article. Viewpoints of MLHIV were compared with those of men whose status was unknown to identify similarities and differences in perspectives about the service delivery-related factors that influence men’s engagement in the HIV continuum of care. Due to the sensitive nature of the study, we were unable to involve participants in data analysis and dissemination. Findings from the qualitative study, which included evidence presented in this manuscript, were shared with stakeholders for input during dissemination workshops.

## Results

Across three sites, 227 adult men participated in the study. Four men recruited to the study chose not to participate; no participants dropped out of the study. The sample contained a higher proportion of men in the older age category (57 men aged 35 to 49 vs. 44 men 25 to 34), and 28 were known to be living with HIV. Table 1 summarizes the distribution of participants across age, roles, interview type, and HIV status.

**Table 1:** Sample distribution.

Type of participant	Number of IDIs		Number of FGDs (4–8 participants per group)		Total number of participants
Age Group	25–34	34–49	25–34	34–49	
Adult men: HIV-status unknown to investigators	19	26	12	16	199 (45 IDIs, 154 in FGDs)
Adult men: living with HIV, in treatment	6	9	-	-	15
Adult men: living with HIV, not in treatment	7	6	-	-	13
**Total adult men participating in the study**					**227**

Abbreviations: FGD, focus groups discussion; IDI, in-depth interview

Overall, we found both costs and benefits related to interactions with the provider. Costs included the fear of unwanted disclosure, actual or anticipated stigma, and the belief that providers were not administering the HIV test properly. These costs were offset by the perceived benefits of social support from the provider and clinical guidance on the treatment journey. Men also identified a plethora of costs linked to the facility itself, separate from interactions with the provider. The layout of the clinic–where clients living with HIV waited or which provider they saw–was felt to compromise the confidentiality of clients’ HIV status. Men also identified wait times, ART stock-outs, and financial costs as barriers. Finally, some men expressed a belief that the formal health system was primarily for women and children, and that clinics were feminine spaces not to be frequented regularly by men.

### Provider-level factors

Perceived costs associated with provider interactions between the individual and a nurse or physician at the facility centered on the following: (1) fear that providers would disclose men’s HIV status and (2) anticipated or enacted stigmatization from providers.

#### Perceived cost: Unwanted disclosure

Men of unknown status were mostly concerned about confidentiality among providers. A man of unknown status described concern that providers would gossip after an HIV diagnosis, saying, “I believe that in this area, the doctors must be a little bit discrete. Discretion, especially when they finish doing the test and they have girlfriends around. That’s where they share the information” (FGD, man of unknown status, 25–34, San Pedro). In contrast, MLHIV who had used HIV related services generally did not perceive a lack of confidentiality in relationships with providers.

#### Perceived cost: Anticipatory or enacted stigma

In general, MLHIV who were in treatment tended to be positive about their interactions and relationships with providers, while men of unknown status or MLHIV not in care more often expressed skepticism of providers’ motives, reception, or skills. For MLHIV not in treatment, negative experiences with staff at health facilities generally related to anticipated or experienced demeaning behavior (generally perceived as a result of their HIV status). For example, a MLHIV in Bouaké described the experience that led him to seek guidance from the Internet and care from traditional healers rather than the formal system:

I condemn above all the behavior of certain health providers. Their reception is disappointing. I prefer to stay at home, I prefer to connect to the internet to search for what I can do. I prefer to go to the women who sell traditional medicines. When you get to the hospital, you feel as if you have failed, being sick… When they discover it is HIV, they give you a weird look. When your back is turned, the staff laughs. I lived it yesterday. I lived it yesterday and it hurt me. (IDI, MLHIV not in treatment, 25–34, Bouaké)

A MLHIV not in treatment described his desire to reengage in care after lapsing, but was afraid to go to the clinic: “If I go back, are they not going to chase me away or say something to me? Because they accepted me in the beginning, and now I have not gone for 8 or 10 months today. If I go back, won’t I have problems?” (IDI, MLHIV not in treatment, 35–49, San Pedro). Men of unknown status echoed similar concerns, anticipating disrespectful treatment or moralizing from providers: “The welcome even in the hospital—they say you are sick, they say that you have sinned, the medical staff even discourages us” (FGD, man of unknown status, 35–49, Abidjan).

#### Perceived cost: Mistrust of providers in administering HIV test

Along with a failure to trust confidentiality and the anticipation of demeaning behavior from providers offering HIV testing services, men questioned the reliability of providers to administer or interpret the test. Men of unknown status were particularly skeptical of the testing process. For example, a man of unknown status in San Pedro lamented: “You see how in Africa our doctors take human life lightly. Today it hurts me that after a doctor tells you, you have HIV, you are forced to go other places two or three times to be sure you really have HIV-AIDS” (FGD, man of unknown status, 25–34, San Pedro). Another said: “I do not doubt the reliability of the test, the test we all know that it is reliable, but sometimes the person who does the test can be wrong” (FGD, man of unknown status, 35–49, Abidjan). Questioning the reliability of the HIV test interpretation negatively modulated the benefit seen in accessing care–both the value in getting tested and the subsequent HIV related care if the test was interpreted as positive, because of the concern of an incorrect HIV positive diagnosis leading to mental distress and care for a condition that they did not have.

Despite these issues both men of unknown status and MLHIV uniformly recommended going to the health facility if diagnosed with HIV.

#### Perceived benefit: Social support

Social support offered through a relationship with the provider was perceived as a benefit for men in the study. MLHIV expressed this benefit more often and in stronger terms than men whose HIV status is unknown. The moment of diagnosis was particularly poignant as men were dealing with the psychological implications of the diagnosis (describing themselves as shocked or stricken) as well as the need to begin a new way of life (characterized as a new way to walk or to live now). These positive experiences were generally described by MLHIV who were successfully engaged in care. Even men who were not yet linked to care anticipated support as a benefit; a MLHIV in Abidjan waiting to begin treatment described support from providers as one of the major benefits of engaging in treatment: “All of that is part of healing: it is not just taking medications, if there are people who are there to advise you and take care of you” (IDI, MLHIV not in treatment, 25–34, Abidjan). MLHIV defined such support in a variety of ways, particularly at the moment of diagnosis to encourage treatment initiation, with one man giving an example in which providers “truly motivated me a lot, gave me hope that one day I can witness the wonders of treatment” (IDI, MLHIV in treatment, 35–49, Abidjan). Having the illness normalized through comparison to other common and less stigmatized illnesses resonated with men. Newly diagnosed men spoke of their encouragement when providers showed them examples of others living with HIV who, in the eyes of participants, appeared to be healthy, although direct social support from other MLHIV in the facility setting was not mentioned as a factor. Men also described additional services from providers that could help living with HIV, including accompanying the client home to support disclosure to partners and regular availability via phone calls outside the office.

#### Perceived benefit: Clinical guidance

The MLHIV in care viewed and valued providers as the primary source of guidance and medical decision-making to maintain health. One MLHIV described the positive relationship as “the doctor is a guide, he is your God on earth” (IDI, MLHIV in treatment, 35–49, Abidjan). One MLHIV on treatment from Bouaké said, “the doctors, I tell myself that they know their work, they know at what time it is right for the person to benefit from treatment” (IDI, MLHIV in treatment, 25–34, Abidjan). The emphasis was on directive communication, with the provider as the authority. Likewise, men of unknown HIV status cited counseling and clinical guidance from providers as a key motivator for going to the facility, if diagnosed. Treatment decision-making was considered, by male participants, to be entirely the domain of providers. As a result, trust and confidence in the providers was essential to maintain engagement in care. While acknowledging the fallibility of providers, participants also expressed a need to maintain trust: “I did not do medical school, so I do not discuss with the doctor. It’s true he can be wrong, but he has finished school, so if the doctor sees something and tells me, it’s up to me to listen to what he told me and to follow” (IDI, man of unknown status, 35–49, Bouaké). In general, men emphasized a need for providers to be competent and knowledgeable about HIV, and they hoped to receive counsel from “someone truly who has the knowledge in this domain, who can truly explain and take his time to explain all of that” (IDI, man of unknown status, 35–49, Bouaké). However, MLHIV identified certain deficits in client-provider communication, such as the use of too many technical terms or not taking time to explain treatment. ART eligibility, viral load, and CD4 count were all concepts that many men, both men of unknown status and MLHIV, were unable to explain.

### Health facility-level factors

Facility level factors played an important role in shaping HIV-related care-seeking behavior among men on multiple levels. A prominent theme was the perception that the layout of the health facility did not adequately protect the privacy of MLHIV or was more suited to women than men. Participants also described some of the perceived costs, such as wait times or having to miss work, frustration of stock-outs of antiretrovirals, and fees collected at the facility even if the medications themselves were free.

#### Perceived cost: Unwanted disclosure

As with potential provider cost, confidentiality was a concern based on the organization and layout of a facility itself. Men described HIV services as usually spatially separated from other health services, increasing the chance of other clients inadvertently suspecting their HIV diagnosis. A man of unknown status in San Pedro described how someone’s presence in a particular waiting area might disclose one’s status and become a barrier to retention:

Because of shame, this person is not following treatment, because at the hospital as they say, there is a bench for those with HIV. When you sit there, you wait for medication, people know that you have HIV. The person has so much fear of this, so much shame, that the person will not go there. The person will not follow treatment (FGD, man of unknown status, 25–34, San Pedro)

Similarly, MLHIV described sometimes seeing acquaintances at the facility and trying slip out before receiving services to avoid being associated with HIV.

#### Perceived cost: Time

The time associated with long waits at the clinic and unavailable services was a substantial cost to some men. Men of unknown status particularly anticipated wait times that would be prohibitive, based on prior experience seeking non-HIV health services. In IDIs and FGDs, men expressed their frustration at the delays and waiting involved in visiting a health facility. For example, a MLHIV in Bouaké recounted a conversation with a nurse in which he inquired about the wait time and was sharply reprimanded: “There is a nurse who told me in Abidjan, ‘Did I infect you? If you do not want to wait, you can leave.’ I said, ‘ma’am, I am sick, that’s why I asked if the doctor is still busy’” (IDI, MLHIV not in treatment, 25–34, Bouaké). Further, both MLHIV and men of unknown status anticipated or described issues with scheduling testing or treatment visits around their work schedules and felt that missing work was a cost they were unable to pay. The potential or actual cost of missing work due to side effects was also mentioned as a barrier to initiating or staying on treatment by MLHIV. In addition to wait times, men cited examples of seeking testing or treatment services and being turned away because the appropriate provider was not available that day. A newly diagnosed MLHIV in Abidjan described an experience of going to a facility to begin treatment, but the provider had not come to the facility: “I cannot take the medication at random, I must have a prescription. Today I had an appointment at the hospital, but they said that the doctor was not coming to the hospital today” (IDI, MLHIV not in treatment, 25–34, Abidjan).

#### Perceived cost: ART stock-outs

Availability of medicine in facilities impacted whether MLHIV were able to initiate treatment or remain adherent. A MLHIV in Abidjan explained that the ART was not always available and stock-outs interrupted his treatment reducing his confidence in the facility. “When I come, [the provider] gives me advice, he tells me to take my medication. I tell him yes I will take the medication, but often when I come there is no medication. So when there’s no medication like that, I am discouraged” (IDI, MLHIV in treatment, 25–34, Abidjan).

#### Perceived cost: Financial

Men of unknown status, in particular, perceived visits to the health facility for any health concern to be expensive, particularly potential clinic fees (even though generally absent for HIV services). When discussing financial costs, the informal health system (traditional care or self-medicating with herbal products) was frequently cited as an alternative (and often preferred) source of healthcare for men, with many opting for concurrent or serial care from both the formal and informal sector. A man of unknown status described that men who opt for the traditional system “think that if they go to the hospital, the costs will be exorbitant. So they prefer to stay in their corners, do their traditional treatments” (FGD, man of unknown status, 25–34, Bouaké). Generally, factors such as distance, convenience, and contextual familiarity were additional factors in keeping men in the informal system reducing actual financial costs and additional barriers to the formal sector. MLHIV who were in treatment tended to critique use of the informal system (including rumors about HIV cures offered by traditional or religious healers). However, MLHIV not in treatment did revert to the traditional system at times, not by preference, but if they felt that access issues prevented them from continuing on ART (for example, difficulty accessing clinics when traveling).

### Perceived cost: Clinics as feminine spaces

Men identified health facilities as a place primarily oriented toward addressing the needs of women and children while viewing the traditional system as sometimes more suited for their own care. This preference was partly explained by the perception of clinics as feminine spaces and partly due to some men’s perceptions that seeking help was an admission of weakness, a characteristic inappropriate for men. In general, men often felt that women were quick to visit the health facility with the slightest of health concerns, whereas they themselves delayed or avoided the formal health system to demonstrate strength. The image of the health facility as a feminine space was more common among men of unknown status who had less experience with clinics than MLHIV.

Table 2 summarizes perceived benefits and costs at the client-provider (interpersonal) and facility levels.

**Table 2:** Factors affecting HIV care seeking (perceived costs and values).

Level	Perceived Cost	Perceived Benefit
Provider-level factors	Unwanted disclosure Anticipatory or enacted stigma Mistrust of providers in administering HIV test	Social support Clinical guidance
Facility-infrastructure level factors	Structural risk of unwanted disclosure Time cost ART stock-outs Financial cost Clinics as feminine spaces	

Table 2 illustrates that the expressed costs of seeking facility-based HIV care considerably outweigh the benefits in the minds of Ivorian men, though the balance is more even at the provider level.

## Discussion

Several key insights emerged in men’s perceptions of, and experiences with, clinicians and clinics in regard to HIV testing and HIV care. Both MLHIV not in treatment and men with unknown HIV status were concerned about mistreatment or confidentiality breaches by providers. Clinic procedures were seen as potentially leading to inadvertent disclosure through being seen at the clinic or in a queue for HIV services. Providers were seen as posing risks for disclosure or enacted stigma to many men, especially those not diagnosed or not yet in care. Provider interactions sometimes had the added psychosocial benefit. This highlights a dichotomy between anticipated negative experiences among men not engaged in HIV care in our study and lived, generally positive, experiences among men engaged in care.

The costs and benefits that men described were consistent with previous literature regarding providers and care settings in other LMIC settings, such as stigmatization, unwanted disclosure, or long wait times and frequent repeat visits [13,24,25]. The benefit of support and clinical guidance or a warm and trusting client-provider relationship intersects with conceptions of trust in health providers identified in other settings: competence, compassion, and confidentiality [26,27]. Here we have expanded understanding of these costs to men and men’s experiences in the context of health care. We chose to focus on the health care provider and facility interactions due to the centrality of these in engaging men in HIV care and the need to understand this dynamic to optimize current service delivery models. Furthermore, we choose to focus on men to identify care considerations for improving engagement in care for men. Some of these factors are likely different between men and women and some differences were even described by participants. However, as a study meant to inform care for men, we did not seek to differentiate or compare costs or value between men and women. In addition, our focus on the care setting and the provider is not meant to minimize other factors that add benefit or costs to accessing care.

A strength of the study was the large sample size with a wide representation of participant characteristics including diversity by age, employment, HIV status, engagement with the continuum of care, and area of Cote d’Ivoire in which they resided. This study also has important limitations. It is possible that the recruitment approach influenced responses; as recruitment was supported by NGOs doing HIV-related work in communities, participants may have been more likely to seek out HIV services or view services positively. The study had a large sample size and was conducted as formative research before the implementation of the current version of Brothers for Life, so we expect there was sufficient variation in the sample with respect to views of HIV services. The research was conducted in areas that were primarily urban, potentially limiting generalization to more rural areas of Côte d’Ivoire. In addition, some of the MLHIV in this study who were not engaged in care had been excluded from ART by treatment policy rather than individual volition. We believe that their perspectives as newly diagnosed men offered valuable insight to perceptions and experiences in accessing HIV testing and navigating the health care system.

By understanding the perceptions of men in weighing the benefits and costs of care seeking, program implementers may better meet the needs of male clients. For example, re-emphasizing the critical importance of maintaining confidentiality among service providers may improve the experience of male clients once they access care. Communication and community-based interventions that specifically incorporate gender norms and gender roles may also more effectively reach men and potentially shift perceptions of health care facilties. If such communication is effective, negative expectations of care experiences may be able to be shifted to more realistic expectations of the clinic experience. Support of men’s testing and care outside of facilities (in spaces considered more suitable by men overall) may also help to improve engagement in care, such as community-centered ART delivery and other differentiated models of care [28,29]. Understanding the perspectives of men is critical to tailoring health communication and clinical services to meet the needs of men. This work adds to the growing body of practical knowledge that can be used to improve engagement with HIV services among men in Cote d’Ivoire.

## Supporting information

S1 File COREQ checklist. COnsolidated criteria for REporting Qualitative research (COREQ) checklist.
(PDF)

S2 File IDI and FGD guide (English). Guides for in-depth interviews and focus group discussions, in English.
(PDF)

S3 File IDI and FGD guide (French). Guides for in-depth interviews and focus group discussions, in French.
(PDF)
