# Supplementary material for: Men’s perceptions of HIV care engagement at the facility- and provider-levels: Experiences in Cote d’Ivoire
Source: PLoS One. 2019 Mar 21;14(3):e0211385. doi: 10.1371/journal.pone.0211385 (PMC6428322; doi:10.1371/journal.pone.0211385)
Supplement: S3 File — Guides for in-depth interviews and focus group discussions, in French. (PDF) [file pone.0211385.s003.pdf]

Entretien individuel approfondi : Hommes adultes

Introduction

(Durée approximative : 1 heure)

Commencez l'entretien par une introduction et le processus de consentement :

Je vous remercie de parler avec moi aujourd'hui. Les informations que vous me donnerez resteront totalement confidentielles et seront utiles pour améliorer les services de santé (en particulier autour de la question du VIH et de la santé sexuelle) pour les hommes.

Les paragraphes suivants donnent des instructions générales sur la conduite de l'entretien.

Les questions ci-dessous sont des points de départ de discussion pour des sujets clés. Il ne s'agit pas d'une série de questions à poser, ni contenir le langage spécifique convenant au mieux à chaque groupe d'individus. Une discussion approfondie sur un sujet particulier est plus utile qu'une discussion superficielle de tous les thèmes inclus dans ce document.

Pour faciliter la participation du répondant à l'entretien, il est important d'établir un contact avec cette personne en posant des questions simples du genre : « Comment allez-vous aujourd'hui ? ».

Nombre de ces questions mettent l'accent sur les perceptions de la santé, des soins de santé et du VIH. Les questions peuvent être orientées vers le point de vue du participant ou vers ce que, selon lui, d'autres hommes comme lui penseraient. Parfois, le fait de décrire ce que les autres pensent permet à un participant d'être plus ouvert par rapport à son propre point de vue.

**GUIDE D'ENTRETIEN APPROFONDI POUR LES PARTICIPANTS DONT L'ENQUETEUR NE CONNAIT PAS (ET NE DEMANDERA PAS) LE STATUT**	
**Thème**	**Questions**
**Information Démographique**	Sans dire votre nom, présentez-vous en donnant votre âge, niveau d'instruction, statut matrimonial, et profession.
**Aspirations**	Qu'est-ce qui est important pour vous dans la vie ? Essayez de penser aux 3-5 choses les plus importantes.<br>***Pour chaque valeur :***<br>Qu'est-ce que [la valeur] signifie pour vous ?<br>Que faites-vous maintenant pour atteindre [valeur] ?<br>Que pensez-vous des autres personnes qui ont ce [valeur] ?<br>Que ferez-vous à l'avenir pour atteindre [valeur] ?<br>Qu'est-ce qui vous donneriez le sentiment que vous avez réussi dans la vie ?
**Sante**	***[Si la santé n'a pas été parmi les 3-5 valeurs les plus importantes].***<br>Comment est-ce que la santé est-elle liée aux valeurs que vous avez énumérées ?<br>Qu'est-ce que la santé signifie pour vous ?<br>Qu'est-ce qu'une personne en bonne santé peut faire ?<br>Qu'est-ce qui permet de dire que quelqu'un n'a pas la santé ?<br>Que faites-vous maintenant pour avoir la santé ?<br>Que ferez-vous à l'avenir pour parvenir à la santé ?<br>Quelles sont vos plus grandes préoccupations ou besoins en matière de santé?

<table>
<tr><td rowspan="2">Sante et Genre</td><td>Question 1</td><td>Question 2</td><td>Question 3</td></tr>
<tr><td>Décrivez un homme qui est en bonne santé.

Comment parait-il ?

Décrire une femme qui est en bonne santé.

Comment parait-elle ?

Quelles sont les différences en ce qui signifie la santé pour les hommes par rapport aux femmes ? Quelles sont les similarités ?</td><td>Qu'est-ce que vous faites pour protéger votre santé et votre bien-être ?

Est-ce que vous prenez des médicaments ou des produits pour maintenir votre sante régulièrement sur le long terme ? Si oui, qu’est-ce que vous prenez et pourquoi ?</td><td>Que font les femmes que vous connaissez pour protéger leur santé et leur bien-être ?

Que pensez-vous de femmes qui prennent des médicaments ou des produits pour maintenir leur sante régulièrement sur le long terme ?</td></tr>
<tr><td rowspan="2">VIH</td><td>Question 1</td><td>Question 2</td><td>Question 3</td></tr>
<tr><td>Qu'est-ce que le VIH signifie pour vous ?

Expliquez plus ce que vous voulez dire.

Comment pensez-vous qu'un homme peut savoir qu'il a le VIH?</td><td>Si vous apprenez qu'un homme que vous connaissez à le VIH, comment réagiriez-vous ?

Qui sont les hommes susceptibles à avoir le VIH ?</td><td>Qu’est-ce qu’un homme devrait faire s’il pense qu’il a une infection sexuellement transmissible ? Qu’est-ce qu’il devrait faire s’il a d’autres problèmes sexuels ? (comme la performance sexuel, etc.)</td></tr>
<tr><td>Test de Dépistage</td><td colspan="3">Qu’est-ce que le dépistage du VIH ?
[Si le participant ne reconnait pas le mot « dépistage », explique-lui que le dépistage c’est un test sanguin pour savoir si une personne à le VIH ou pas]
Qui devrait se faire dépister pour le VIH ?
Pourquoi un homme voudrait se faire dépister pour le VIH ? Pourquoi un homme ne voudrait pas se faire dépister pour le VIH ? Pourquoi une femme voudrait se faire dépister pour le VIH ? Pourquoi une femme ne voudrait pas se faire dépister pour le VIH ?
S’on dit que vous n’avez le VIH, qu'est-ce que ça veut dire ? Comment réagiriez-vous ? Et s’on dit que vous n’avez pas le VIH qu’est-ce que ça veut dire ? Comment réagiriez-vous ?</td></tr>
<tr><td rowspan="2">Tester/Traiter</td><td colspan="3">Scenario</td></tr>
<tr><td colspan="3">Paul est un homme avec une femme et 2 enfants qui travaille dans une société. Il est allé pour un faire son test VIH aujourd'hui et on lui dit qu’il a le VIH. Que pensez-vous qui se passe à travers sa tête ? Que pensez-vous qu'il pourrait faire ? Avec qui pensez-vous qu'il pourrait parler ?
Le conseiller communautaire lui demande de retourner voir le médecin. Que penseriez-vous si vous étiez Paul ? Est-ce que Paul devrait retourner voir le</td></tr>
</table>

	médecin ? Pourquoi ou pourquoi pas ?<br>**Paul va chez le médecin et on lui donne des médicaments contre le VIH à prendre chaque jour avant de se coucher.** Doit-il les prendre ? Pourquoi ou pourquoi pas ?
**Traitement**	Qu'est-ce que vous savez du traitement pour le VIH ? (raisons pour faire le traitement, difficultés, etc.)

Entretien individuel approfondi : Hommes adultes séropositifs

Introduction

(Durée approximative : 1 heure)

Commencez l'entretien par une introduction et le processus de consentement :

Je vous remercie de parler avec moi aujourd'hui. Les informations que vous me donnerez resteront totalement confidentielles et seront utiles pour améliorer les services de santé (en particulier autour de la question du VIH et de la santé sexuelle) pour les hommes.

Les paragraphes suivants donnent des instructions générales sur la conduite de l'entretien.

Les questions ci-dessous sont des points de départ de discussion pour des sujets clés. Il ne s'agit pas d'une série de questions à poser, ni contenir le langage spécifique convenant au mieux à chaque groupe d'individus. Une discussion approfondie sur un sujet particulier est plus utile qu'une discussion superficielle de tous les thèmes inclus dans ce document.

Pour faciliter la participation du répondant à l'entretien, il est important d'établir un contact avec cette personne en posant des questions simples du genre : « Comment allez-vous aujourd'hui ? ».

Nombre de ces questions mettent l'accent sur les perceptions de la santé, des soins de santé et du VIH. Les questions peuvent être orientées vers le point de vue du participant ou vers ce que, selon lui, d'autres hommes comme lui penseraient. Parfois, le fait de décrire ce que les autres pensent permet à un participant d'être plus ouvert par rapport à son propre point de vue.

**GUIDE D'ENTRETIEN INDIVIDUEL APPROFONDI AVEC LES HOMMES SEROPOSITIFS**			
**Theme**	**Questions**		
**Information Démographique**	Sans dire votre nom, présentez-vous en donnant votre âge, niveau d'instruction, statut matrimonial, et profession.		
**Aspirations**	Qu'est-ce que vous valorisez le plus dans la vie ? Essayez de penser aux 3-5 choses le plus importantes.<br>***Pour chaque valeur :***<br>Qu'est-ce que [la valeur] signifie pour vous ?<br>Que faites-vous maintenant pour atteindre [valeur] ?<br>Que pensez-vous d'autres qui ont [valeur] ?<br>Que ferez-vous à l'avenir pour atteindre [valeur] ?<br>Qu'est-ce qui vous donneriez le sentiment que vous avez réussi dans la vie ?		
**Santé**	***[Si la santé n'a pas été parmi les 3-5 valeurs les plus importantes].***<br>Comment est-ce que la santé est-elle liée aux valeurs que vous avez énumérées ?<br>Qu'est-ce que la santé signifie pour vous ?<br>Qu'est-ce qu'une personne en bonne santé peut faire ?<br>Qu'est-ce qui permet de dire que quelqu'un n'a pas la santé ?<br>Que faites-vous maintenant pour avoir la santé ?<br>Que ferez-vous à l'avenir pour parvenir à la santé ?<br>Quelles sont vos plus grandes préoccupations ou besoins en matière de santé?<br>Chez qui est-ce que vous prenez des conseils ? (des amis, des guérisseurs traditionnels/spirituels, des agents de santé, etc).		
**Santé et Genre**	Question 1	Question 2	Question 3

<table>
<tr><td></td><td>Décrivez un homme qui est en bonne santé.

Comment parait-il ?

Décrire une femme qui est en bonne santé.

Comment parait-elle ?

Quelles sont les différences en ce qui signifie la santé pour les hommes par rapport aux femmes ? Quelles sont les similarités ?</td><td colspan="2">Qu'est-ce que les hommes comme vous font pour protéger leur santé et leur bien-être ?</td><td>Que font les femmes dans votre communauté pour protéger leur santé et leur bien-être ?</td></tr>
<tr><td>HIV</td><td colspan="4">Qu'est-ce que le VIH signifie pour vous ? Expliquez plus ce que vous voulez dire. Est-ce que vous avez partagé votre statut sérologique avec quelqu’un ? Si oui, qui ? Décrit l’expérience de partage. Si non, qu’est-ce qui vous a empêché de partager votre statut sérologique ?</td></tr>
<tr><td>HIV Testing</td><td colspan="4">Comment est-ce que vous avez appris que vous avez le VIH ?
Pour quoi est-ce que vous avez fait le test ?
Décrit ce qui s’est passé après que vous avez reçu les résultats du dépistage.</td></tr>
<tr><td>*Hommes séropositifs</td><td colspan="4">Quel traitement recevez-vous actuellement ? Quel est l'objectif de votre traitement ? Comment savez-vous si le traitement fonctionne ou pas?</td></tr>
<tr><td>Connaissance sur le traitement VIH</td><td colspan="4">Quels sont les traitements disponibles pour le VIH ici ? Pour qui sont-ils disponibles ?
Quel est le but du traitement ?
Avez-vous déjà entendu parler du taux CD4 ? Si oui, expliquer ce que cela signifie pour vous ?
Avez-vous déjà entendu parler de la charge virale ? Si oui, expliquer ce que cela signifie pour vous ?
Et la suppression virale, qu’est-ce que cela signifie pour vous ?</td></tr>
<tr><td rowspan="2">Tester/Traiter</td><td colspan="2">Question 1</td><td colspan="2">Question 2</td></tr>
<tr><td colspan="2">Certaines personnes vivant avec le VIH bénéficient d'un traitement et d'autres ne le font pas. Que penses-tu de cela ? Qu'est-ce que le traitement du VIH signifie pour vous?</td><td colspan="2">Que penseriez-vous si toutes les personnes avec le VIH recevez systématiquement le traitement ? Que pensera la population en général si tous les personnes avec le VIH recevez systématiquement le traitement ?</td></tr>
</table>

***HIV Positive**	***Si le participant est sous traitement antirétroviral :*** Qu'est-ce qui vous a motivé pour commencer le traitement ?<br>***Si le participant n'est pas sous traitement antirétroviral :*** Voulez-vous commencer le traitement ? Pourquoi ou pourquoi pas ?<br>Qu'est-ce qui vous motive (ou motive à d'autres comme vous) de rester sous traitement ?<br>Qu'est-ce qui vous empêche (ou empêche à d'autres personnes comme vous) de rester sous traitement ?<br>Qu'est-ce qui pourrait vous amener (ou entrainer a d'autres comme vous à abandonner le traitement ?<br>Qu'aimeriez-vous savoir d'autre sur le traitement VIH ?

Groupe de discussion : Hommes adultes
Introduction
(Durée d'environ 1H30)

Les paragraphes suivants donnent des instructions générales sur la conduite de l'entretien.

Les questions ci-dessous sont des points de départ de discussion pour des sujets clés. Il ne s'agit pas d'une série de questions à poser, ni contenir le langage spécifique convenant au mieux à chaque groupe d'individus. Une discussion approfondie sur un sujet particulier est plus utile qu'une discussion superficielle de tous les thèmes inclus dans ce document.

Pour faciliter la participation du répondant à l'entretien, il est important d'établir un contact avec cette personne en posant des questions simples du genre : « Comment allez-vous aujourd'hui ? ».
Nombre de ces questions mettent l'accent sur les perceptions de la santé, des soins de santé et du VIH. Les questions peuvent être orientées vers le point de vue du participant ou vers ce que, selon lui, d'autres hommes comme lui penseraient. Parfois, le fait de décrire ce que les autres pensent permet à un participant d'être plus ouvert par rapport à son propre point de vue.

L'objectif est d'identifier et de décrire les normes communautaires et les thèmes autour de ces sujets et pas de comprendre les nuances de chaque membre du groupe individuellement.

Au début du groupe de discussion
Commencez par remercier les participants :

"Je vous remercie de parler avec moi aujourd'hui. Les informations que vous fournissez resteront confidentielles et seront utiles pour améliorer les services de santé pour les hommes (en particulier les services de santé pour le VIH et la santé sexuelle). "

Chaque sujet / question devrait conduire à une discussion de groupe. Les points de vue du groupe sont importants, beaucoup plus que les points de vues de chaque individu.

**GUIDE DE GROUPE DE DISCUSSION POUR LES PARTICIPANTS DONT L'ENQUETEUR NE CONNAIT PAS (ET NE DEMANDERA PAS) LE STATUT**	
**Thème**	**Questions**
**Information Démographique**	Sans dire votre nom, présentez-vous en donnant votre âge, niveau d'instruction, statut matrimonial, et profession.
**Aspirations**	Qu'est-ce que les hommes dans votre communauté valorisent le plus dans la vie ?<br>Essayez de penser aux 3-5 choses les plus importantes.<br>***Pour chaque valeur :***<br>Qu'est-ce que [la valeur] signifie dans la communauté ?<br>Comment est-ce que [la valeur] est atteint ?<br>Qu'est-ce qui fait qu'un homme a réussi dans la vie ?
**Sante et Genre : Hommes**	***[Si la santé n'a pas été parmi les 3-5 valeurs les plus importantes].***<br>Comment est-ce que la santé est-elle liée aux valeurs les plus importantes pour les hommes dans votre communauté?<br>Qu'est-ce que la santé signifie pour vous et comment faire pour la préserver ?<br>Quelles sont les maladies courantes dont les hommes souffrent ?

<table>
<tr><td></td><td colspan="3">Que font les hommes pour ne pas attraper les maladies ?
Que font les hommes pour traiter les maladies ?</td></tr>
<tr><td>Sante et Genre : Femmes</td><td colspan="3">Et pour les femmes, qu’est-ce que la sante signifie pour elles ?
Quelles sont les maladies courantes dont les femmes souffrent ?
Que font les femmes pour ne pas attraper des maladies ?
Que font les femmes pour traiter les maladies ?</td></tr>
<tr><td rowspan="2">VIH</td><td>Question 1</td><td>Question 2</td><td>Question 3</td></tr>
<tr><td>Qu'est-ce que le VIH signifie pour les hommes dans votre communauté?

Expliquez plus ce que vous voulez dire.

Quels hommes sont plus à risque d’attraper le VIH ?</td><td>Comment pensez-vous qu'un homme peut savoir qu'il a le VIH?</td><td>Si un homme a le VIH, doit-il informer sa femme ? Pourquoi ou pourquoi pas ?

Doit-il informer sa petite amie ? Pourquoi ou pourquoi pas ?</td></tr>
<tr><td>Test de Dépistage</td><td colspan="3">Qu’est-ce que le dépistage du VIH ?
[Si les participants ne reconnaissent pas le mot « dépistage », explique-les que le dépistage c’est un test sanguin pour savoir si une personne à le VIH ou pas]
Qui devrait se faire tester pour le VIH ?
Pourquoi un homme voudrait se faire dépister pour le VIH ? Pourquoi un homme ne voudrait pas se faire dépister pour le VIH ?
S’on dit que vous n’avez le VIH, qu'est-ce que ça veut dire ? Comment réagiriez-vous ? Et s’on dit que vous n’avez pas le VIH qu’est-ce que ça veut dire ? Comment réagiriez-vous ?</td></tr>
<tr><td rowspan="2">Tester/Traiter</td><td colspan="3">Scenario</td></tr>
<tr><td colspan="3">Paul est un homme avec une femme et 2 enfants qui travaille dans une société. Il est allé pour un faire son test VIH aujourd'hui et on lui dit qu’il a le VIH. Que pensez-vous qui se passe à travers sa tête ? Que pensez-vous qu'il pourrait faire ? Avec qui pensez-vous qu'il pourrait parler ?
Le conseiller communautaire lui demande de retourner voir le médecin. Que penseriez-vous si vous étiez Paul ? Est-ce que Paul devrait retourner voir le médecin ? Pourquoi ou pourquoi pas ? Qu’est-ce que le groupe pense ?
Paul va chez le médecin et on lui donne des médicaments contre le VIH à prendre chaque jour avant de se coucher. Doit-il les prendre ? Pourquoi ou pourquoi pas ?
Connaissez-vous quelqu'un avec le VIH ?
Savez-vous s’ils reçoivent un traitement ? Si non : Que pensez-vous de cela ? Si oui : Est-ce que vous pensez que le traitement est en train de lui aider ?</td></tr>
<tr><td>Connaissance sur le traitement VIH</td><td colspan="3">Quels sont les traitements disponibles pour le VIH ici ? Pour qui sont-ils disponibles ?
Quel est le but du traitement ?
Pensez-vous que le traitement doit être offert à tous les personnes séropositives ? Pourquoi ou pourquoi pas ?
Qu’est-ce que vous savez des tests sanguins pour les personnes séropositives ?</td></tr>
</table>

	Qu'est-ce que c'est que le taux de CD4 ? Qu'est-ce que c'est que la charge virale ? Qu'est-ce que c'est la suppression virale ?
